# Poly[[μ-1,4-anhydro­erythritolato-di-μ-aqua-sodium(I)] monohydrate]

**DOI:** 10.1107/S1600536808039640

**Published:** 2008-11-29

**Authors:** Tobias Kerscher, Patrick Zeller, Peter Mayer, Peter Klüfers

**Affiliations:** a Ludwig-Maximilians-Universität, Department Chemie, Butenandtstrasse 5–13, 81377 München, Germany

## Abstract

In the title compound, {[Na(C_4_H_7_O_3_)(H_2_O)_2_]·H_2_O}_*n*_, the sodium ion is octa­hedrally coordinated by two bridging 1,4-anhydro­erythritolate ligands, unexpectedly coordinated by the ring oxygen and four water ligands. This bonding pattern leads to one-dimensional anti­tactical polymeric chains along [010]. One of the exocyclic O atoms of the anhydro­erythritolate group is an acceptor in four hydrogen bonds, giving further evidence that it is deprotonated.

## Related literature

For the neutral 1,4-anhydroerythritole as a coordination ligand on sodium with either the hydoxyl groups coordinating sodium or a mixed coordination by both the ring oxygen and the hydroxyl groups, see: Ballard *et al.* (1974 ▶, 1976 ▶). For puckering parameters, see: Cremer & Pople (1975 ▶).
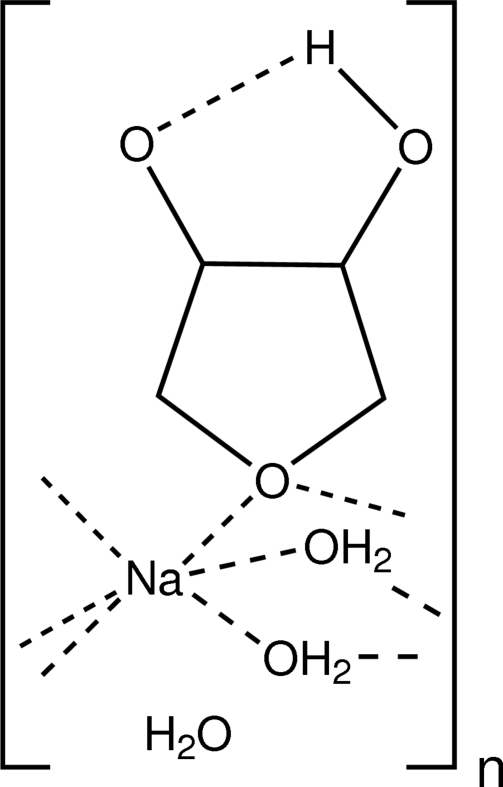


## Experimental

### 

#### Crystal data

[Na(C_4_H_7_O_3_)(H_2_O)_2_]·H_2_O
*M* *_r_* = 180.13
Monoclinic, 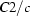

*a* = 23.155 (6) Å
*b* = 6.0900 (16) Å
*c* = 14.543 (5) Å
β = 127.678 (17)°
*V* = 1623.1 (9) Å^3^
*Z* = 8
Mo *K*α radiation
μ = 0.18 mm^−1^
*T* = 200 (2) K
0.23 × 0.20 × 0.10 mm

#### Data collection

Oxford Diffraction XCalibur diffractometer
Absorption correction: none
6310 measured reflections
1699 independent reflections
1088 reflections with *I* > 2σ(*I*)
*R*_int_ = 0.044

#### Refinement

*R*[*F* ^2^ > 2σ(*F* ^2^)] = 0.032
*wR*(*F* ^2^) = 0.086
*S* = 0.95
1699 reflections
118 parameters
9 restraints
H atoms treated by a mixture of independent and constrained refinement
Δρ_max_ = 0.20 e Å^−3^
Δρ_min_ = −0.24 e Å^−3^

### 

Data collection: *CrysAlis CCD* (Oxford Diffraction, 2006 ▶); cell refinement: *CrysAlis RED* (Oxford Diffraction, 2006 ▶); data reduction: *CrysAlis RED*; program(s) used to solve structure: *SHELXS97* (Sheldrick, 2008 ▶); program(s) used to refine structure: *SHELXL97* (Sheldrick, 2008 ▶); molecular graphics: *ORTEPIII* (Burnett & Johnson, 1996 ▶); software used to prepare material for publication: *SHELXL97*.

## Supplementary Material

Crystal structure: contains datablocks I, global. DOI: 10.1107/S1600536808039640/fi2067sup1.cif

Structure factors: contains datablocks I. DOI: 10.1107/S1600536808039640/fi2067Isup2.hkl

Additional supplementary materials:  crystallographic information; 3D view; checkCIF report

## Figures and Tables

**Table 1:** Hydrogen-bond geometry (Å, °)

*D*—H⋯*A*	*D*—H	H⋯*A*	*D*⋯*A*	*D*—H⋯*A*
O2—H2⋯O3	0.82	1.85	2.4367 (17)	127
O4—H41⋯O3^i^	0.839 (14)	1.939 (15)	2.7706 (17)	170.9 (17)
O4—H42⋯O3^ii^	0.883 (14)	1.827 (14)	2.7078 (19)	174.5 (18)
O6—H61⋯O2^ii^	0.877 (14)	1.940 (14)	2.813 (2)	173.1 (18)
O6—H62⋯O5^iii^	0.859 (14)	1.838 (14)	2.6903 (18)	171.0 (18)
O5—H51⋯O3^i^	0.842 (15)	1.825 (15)	2.6653 (18)	176 (2)
O5—H52⋯O2^iv^	0.828 (14)	2.131 (15)	2.958 (2)	177 (2)

Symmetry codes: (i) 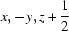; (ii) 
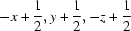
; (iii) 
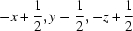
; (iv) 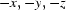.

**Fig. 1.**
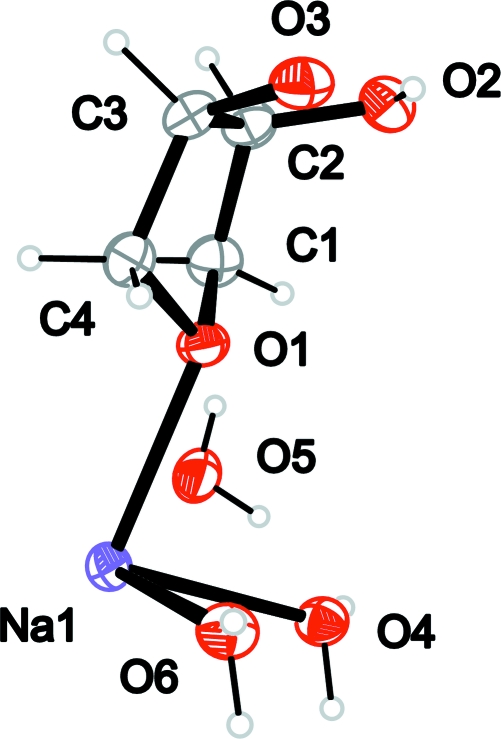
The molecular structure of the title compound, with atom labels and anisotropic displacement ellipsoids (drawn at 50% probability level) for non-H atoms.

**Fig. 2.**
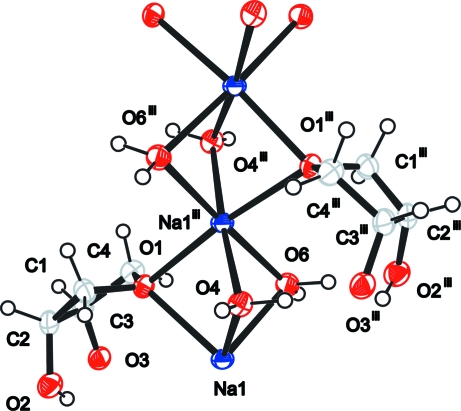
Part of the antitactical polymer-like chain structure of the title compound along [010]. Symmetry code: (iii) -x + 1/2, y - 1/2, -z + 1/2.

## supplementary crystallographic information

### Comment

The title compound was obtained as a byproduct in a reaction involving sodium hydroxide, anhydroerytritole and iron(II) chloride. .

Sodium is octahedrically coordinated by bridging anhydroerythritolate and water ligands. The anhydroerythritolate coordinates the sodium unexpectedly by its ring oxygen atom while the alkoxide group is stabilized by intramolecular hydrogen bonding from the hydroxyl group. The anhydroerythitolate contains a five-membered ring containing O1, C1, C2, C3 and C4 which can be described according to Cremer & Pople (1975) by the puckering parameters q_2_ = 0.3903 Å and Φ_2_ = 180.9772. The closest pucker descriptor is an envelope *E*_O1_.

The bridging ligands lead to a linear chain-like structure along [010] which resembles an antitactical polymer well known from organic chemistry.

### Experimental

The title compound was obtained as a byproduct by the reaction of 40 mMol anhydroerytritol with 160 mMol sodiumhydoxide in 15 mL water at room temperature.

Upon standing for about 14 days at room temperature, colorless platelets of the title compound crystalized from the solution.

### Refinement

Carbon hydrogen atoms and hydoxide hydrogen atoms were calculated in ideal geometry with U(H)=1.2*U(C) for all C-bound hydrogen atoms and U(H)=1.5*U(O) for the hydroxide hydrogen atom. The water-bound hydrogen atoms were found from the difference map, the O-H distances were fixed to 0.84 Å and the H-H distances within the water molecules were fixed to 1.36 Å.

### Crystal data

**Table:**

[Na(C_4_H_7_O_3_)(H_2_O)_2_]·H_2_O_1_	*F*_000_ = 768
*M**_r_* = 180.13	*D*_x_ = 1.474 Mg m^−^^3^
Monoclinic, *C*2/*c*	Mo *K*α radiation λ = 0.71073 Å
Hall symbol: -C 2yc	Cell parameters from 2600 reflections
*a* = 23.155 (6) Å	θ = 3.8–26.5º
*b* = 6.0900 (16) Å	µ = 0.18 mm^−^^1^
*c* = 14.543 (5) Å	*T* = 200 (2) K
β = 127.678 (17)º	Platelet, colourless
*V* = 1623.1 (9) Å^3^	0.23 × 0.20 × 0.10 mm
*Z* = 8	

### Data collection

**Table:**

Oxford Diffraction KappaCCD diffractometer	1088 reflections with *I* > 2σ(*I*)
Radiation source: fine-focus sealed tube	*R*_int_ = 0.044
Monochromator: graphite	θ_max_ = 26.5º
*T* = 200(2) K	θ_min_ = 4.2º
ω scans	*h* = −21→28
Absorption correction: none	*k* = −7→6
6310 measured reflections	*l* = −17→18
1699 independent reflections	

### Refinement

**Table:**

Refinement on *F*^2^	Secondary atom site location: difference Fourier map
Least-squares matrix: full	Hydrogen site location: inferred from neighbouring sites
*R*[*F*^2^ > 2σ(*F*^2^)] = 0.032	H atoms treated by a mixture of independent and constrained refinement
*wR*(*F*^2^) = 0.086	*w* = 1/[σ^2^(*F*_o_^2^) + (0.0484*P*)^2^] where *P* = (*F*_o_^2^ + 2*F*_c_^2^)/3
*S* = 0.95	(Δ/σ)_max_ < 0.001
1699 reflections	Δρ_max_ = 0.20 e Å^−^^3^
118 parameters	Δρ_min_ = −0.24 e Å^−^^3^
9 restraints	Extinction correction: none
Primary atom site location: structure-invariant direct methods	

### Special details

**Table:**

Refinement. Carbon hydrogen atoms and hydroxide hydrogen atoms were calculated in ideal geometry with U(H)=1.2*U(C) for all C-bound hydrogen atoms and U(H)=1.5*U(O) for the hydroxide hydrogen atom. The water-bound hydrogen atoms were found from the difference map, the O-H distances were restrained to 0.84 Å and the H-H distances within the water molecules were restrained to 1.36 Å.

### Fractional atomic coordinates and isotropic or equivalent isotropic displacement parameters (Å^2^)

**Table:**

	*x*	*y*	*z*	*U*_iso_*/*U*_eq_	
Na1	0.24809 (3)	0.43051 (10)	0.24000 (5)	0.0238 (2)	
O1	0.16736 (6)	0.16529 (17)	0.09168 (9)	0.0238 (3)	
O2	0.05729 (6)	−0.21567 (19)	−0.03931 (10)	0.0322 (3)	
H2	0.0846	−0.2907	−0.0445	0.048*	
O3	0.14255 (6)	−0.19459 (18)	−0.08460 (9)	0.0295 (3)	
O4	0.24091 (7)	0.19528 (18)	0.36644 (10)	0.0261 (3)	
H41	0.2075 (8)	0.196 (3)	0.3733 (15)	0.039*	
H42	0.2796 (8)	0.222 (3)	0.4385 (13)	0.039*	
O6	0.34457 (6)	0.18090 (18)	0.30154 (10)	0.0285 (3)	
H61	0.3781 (9)	0.212 (3)	0.3752 (12)	0.043*	
H62	0.3710 (10)	0.123 (3)	0.2850 (14)	0.043*	
C1	0.09012 (9)	0.1596 (3)	0.03391 (15)	0.0288 (4)	
H11	0.0686	0.3080	0.0067	0.035*	
H12	0.0801	0.1041	0.0868	0.035*	
C2	0.05911 (9)	0.0052 (3)	−0.06810 (14)	0.0282 (4)	
H1	0.0097	0.0547	−0.1367	0.034*	
C3	0.11567 (9)	0.0160 (3)	−0.09473 (14)	0.0272 (4)	
H3	0.0922	0.0765	−0.1742	0.033*	
C4	0.17244 (9)	0.1743 (3)	−0.00250 (14)	0.0284 (4)	
H43	0.2218	0.1304	0.0251	0.034*	
H44	0.1626	0.3251	−0.0343	0.034*	
O5	0.06903 (7)	0.4653 (2)	0.23361 (12)	0.0439 (4)	
H51	0.0915 (10)	0.384 (3)	0.2926 (14)	0.066*	
H52	0.0326 (9)	0.400 (3)	0.1783 (15)	0.066*	

### Atomic displacement parameters (Å^2^)

**Table:**

	*U*^11^	*U*^22^	*U*^33^	*U*^12^	*U*^13^	*U*^23^
Na1	0.0278 (4)	0.0183 (4)	0.0225 (4)	−0.0002 (3)	0.0140 (3)	−0.0003 (3)
O1	0.0232 (7)	0.0270 (6)	0.0190 (6)	−0.0019 (5)	0.0116 (5)	−0.0015 (5)
O2	0.0308 (7)	0.0304 (7)	0.0336 (7)	−0.0053 (5)	0.0188 (6)	−0.0022 (5)
O3	0.0292 (7)	0.0322 (7)	0.0242 (7)	0.0002 (5)	0.0149 (6)	−0.0048 (5)
O4	0.0268 (7)	0.0304 (7)	0.0229 (7)	−0.0016 (6)	0.0160 (6)	−0.0025 (5)
O6	0.0269 (7)	0.0299 (7)	0.0281 (7)	0.0016 (5)	0.0165 (6)	−0.0036 (5)
C1	0.0238 (10)	0.0310 (10)	0.0292 (10)	0.0037 (7)	0.0149 (8)	0.0005 (8)
C2	0.0202 (9)	0.0307 (10)	0.0232 (9)	0.0007 (8)	0.0079 (8)	0.0013 (8)
C3	0.0282 (9)	0.0310 (10)	0.0180 (9)	0.0006 (8)	0.0119 (8)	0.0017 (7)
C4	0.0332 (10)	0.0290 (10)	0.0248 (10)	−0.0031 (8)	0.0186 (9)	0.0014 (7)
O5	0.0361 (8)	0.0447 (9)	0.0349 (8)	−0.0085 (6)	0.0136 (7)	0.0117 (6)

### Geometric parameters (Å, °)

**Table:**

Na1—O4^i^	2.3544 (13)	O4—H42	0.883 (14)
Na1—O6	2.3787 (14)	O6—Na1^ii^	2.3893 (14)
Na1—O6^i^	2.3893 (14)	O6—H61	0.877 (14)
Na1—O1	2.4139 (13)	O6—H62	0.859 (14)
Na1—O4	2.4155 (14)	C1—C2	1.516 (2)
Na1—O1^i^	2.4517 (14)	C1—H11	0.9900
Na1—Na1^i^	3.0550 (8)	C1—H12	0.9900
Na1—Na1^ii^	3.0550 (8)	C2—C3	1.578 (2)
O1—C1	1.438 (2)	C2—H1	1.0000
O1—C4	1.445 (2)	C3—C4	1.517 (2)
O1—Na1^ii^	2.4517 (14)	C3—H3	1.0000
O2—C2	1.417 (2)	C4—H43	0.9900
O2—H2	0.8200	C4—H44	0.9900
O3—C3	1.394 (2)	O5—H51	0.842 (15)
O4—Na1^ii^	2.3544 (13)	O5—H52	0.828 (14)
O4—H41	0.839 (14)		
			
?···?	?		
			
O4^i^—Na1—O6	103.35 (5)	Na1^ii^—O4—H41	124.1 (12)
O4^i^—Na1—O6^i^	80.67 (5)	Na1—O4—H41	125.9 (13)
O6—Na1—O6^i^	174.07 (3)	Na1^ii^—O4—H42	117.9 (12)
O4^i^—Na1—O1	101.92 (5)	Na1—O4—H42	107.6 (12)
O6—Na1—O1	86.84 (5)	H41—O4—H42	101.5 (14)
O6^i^—Na1—O1	96.65 (5)	Na1—O6—Na1^ii^	79.69 (4)
O4^i^—Na1—O4	172.80 (5)	Na1—O6—H61	104.5 (12)
O6—Na1—O4	79.65 (5)	Na1^ii^—O6—H61	115.6 (13)
O6^i^—Na1—O4	95.86 (5)	Na1—O6—H62	145.5 (12)
O1—Na1—O4	84.69 (5)	Na1^ii^—O6—H62	110.5 (13)
O4^i^—Na1—O1^i^	85.17 (5)	H61—O6—H62	100.4 (15)
O6—Na1—O1^i^	90.22 (5)	O1—C1—C2	105.23 (13)
O6^i^—Na1—O1^i^	85.75 (5)	O1—C1—H11	110.7
O1—Na1—O1^i^	172.78 (4)	C2—C1—H11	110.7
O4—Na1—O1^i^	88.30 (5)	O1—C1—H12	110.7
O4^i^—Na1—Na1^i^	51.06 (3)	C2—C1—H12	110.7
O6—Na1—Na1^i^	129.44 (5)	H11—C1—H12	108.8
O6^i^—Na1—Na1^i^	50.00 (3)	O2—C2—C1	112.42 (14)
O1—Na1—Na1^i^	135.52 (4)	O2—C2—C3	106.92 (13)
O4—Na1—Na1^i^	122.01 (4)	C1—C2—C3	104.28 (13)
O1^i^—Na1—Na1^i^	50.56 (3)	O2—C2—H1	111.0
O4^i^—Na1—Na1^ii^	137.46 (4)	C1—C2—H1	111.0
O6—Na1—Na1^ii^	50.31 (3)	C3—C2—H1	111.0
O6^i^—Na1—Na1^ii^	129.01 (4)	O3—C3—C4	113.69 (14)
O1—Na1—Na1^ii^	51.66 (3)	O3—C3—C2	108.65 (13)
O4—Na1—Na1^ii^	49.30 (3)	C4—C3—C2	102.13 (13)
O1^i^—Na1—Na1^ii^	121.82 (4)	O3—C3—H3	110.7
Na1^i^—Na1—Na1^ii^	170.73 (4)	C4—C3—H3	110.7
C1—O1—C4	103.87 (12)	C2—C3—H3	110.7
C1—O1—Na1	123.05 (9)	O1—C4—C3	106.58 (13)
C4—O1—Na1	110.75 (9)	O1—C4—H43	110.4
C1—O1—Na1^ii^	121.20 (10)	C3—C4—H43	110.4
C4—O1—Na1^ii^	119.37 (9)	O1—C4—H44	110.4
Na1—O1—Na1^ii^	77.78 (4)	C3—C4—H44	110.4
C2—O2—H2	109.7	H43—C4—H44	108.6
Na1^ii^—O4—Na1	79.64 (4)	H51—O5—H52	109.6 (19)
			
O4^i^—Na1—O1—C1	−98.09 (11)	Na1^i^—Na1—O4—Na1^ii^	−175.97 (5)
O6—Na1—O1—C1	158.92 (11)	O4^i^—Na1—O6—Na1^ii^	−141.91 (5)
O6^i^—Na1—O1—C1	−16.27 (12)	O1—Na1—O6—Na1^ii^	−40.40 (4)
O4—Na1—O1—C1	79.03 (12)	O4—Na1—O6—Na1^ii^	44.77 (4)
Na1^i^—Na1—O1—C1	−52.67 (13)	O1^i^—Na1—O6—Na1^ii^	133.00 (5)
Na1^ii^—Na1—O1—C1	119.44 (12)	Na1^i^—Na1—O6—Na1^ii^	167.97 (6)
O4^i^—Na1—O1—C4	25.43 (10)	C4—O1—C1—C2	41.42 (15)
O6—Na1—O1—C4	−77.55 (10)	Na1—O1—C1—C2	168.00 (9)
O6^i^—Na1—O1—C4	107.26 (10)	Na1^ii^—O1—C1—C2	−96.32 (14)
O4—Na1—O1—C4	−157.45 (10)	O1—C1—C2—O2	90.07 (16)
Na1^i^—Na1—O1—C4	70.85 (11)	O1—C1—C2—C3	−25.39 (17)
Na1^ii^—Na1—O1—C4	−117.04 (10)	O2—C2—C3—O3	1.87 (17)
O4^i^—Na1—O1—Na1^ii^	142.47 (5)	C1—C2—C3—O3	121.13 (14)
O6—Na1—O1—Na1^ii^	39.48 (4)	O2—C2—C3—C4	−118.55 (14)
O6^i^—Na1—O1—Na1^ii^	−135.70 (5)	C1—C2—C3—C4	0.71 (16)
O4—Na1—O1—Na1^ii^	−40.41 (4)	C1—O1—C4—C3	−41.55 (15)
Na1^i^—Na1—O1—Na1^ii^	−172.11 (6)	Na1—O1—C4—C3	−175.52 (10)
O6—Na1—O4—Na1^ii^	−45.63 (4)	Na1^ii^—O1—C4—C3	97.14 (13)
O6^i^—Na1—O4—Na1^ii^	138.29 (5)	O3—C3—C4—O1	−92.61 (16)
O1—Na1—O4—Na1^ii^	42.12 (4)	C2—C3—C4—O1	24.23 (16)
O1^i^—Na1—O4—Na1^ii^	−136.17 (5)		

Symmetry codes: (i) −*x*+1/2, *y*+1/2, −*z*+1/2; (ii) −*x*+1/2, *y*−1/2, −*z*+1/2.

### Hydrogen-bond geometry (Å, °)

**Table:**

*D*—H···*A*	*D*—H	H···*A*	*D*···*A*	*D*—H···*A*
O2—H2···O3	0.82	1.85	2.4367 (17)	127
O4—H41···O3^iii^	0.839 (14)	1.939 (15)	2.7706 (17)	170.9 (17)
O4—H42···O3^i^	0.883 (14)	1.827 (14)	2.7078 (19)	174.5 (18)
O6—H61···O2^i^	0.877 (14)	1.940 (14)	2.813 (2)	173.1 (18)
O6—H62···O5^ii^	0.859 (14)	1.838 (14)	2.6903 (18)	171.0 (18)
O5—H51···O3^iii^	0.842 (15)	1.825 (15)	2.6653 (18)	176 (2)
O5—H52···O2^iv^	0.828 (14)	2.131 (15)	2.958 (2)	177 (2)

Symmetry codes: (iii) *x*, −*y*, *z*+1/2; (i) −*x*+1/2, *y*+1/2, −*z*+1/2; (ii) −*x*+1/2, *y*−1/2, −*z*+1/2; (iv) −*x*, −*y*, −*z*.
